# Biologic Therapy in Pregnant Women with Plaque Psoriasis: A Narrative Review of Maternal and Fetal Safety Considerations

**DOI:** 10.3390/ph19091467

**Published:** 2026-09-16

**Authors:** Shilpa Nagendra, Emmanouil Karampinis

**Affiliations:** 1 Faculty of Life Sciences and Education, University of South Wales, Cardiff NP20 2BP, UK; drshilpabcg@gmail.com; 2 Department of Dermatology, Faculty of Medicine, School of Health Sciences, University General Hospital of Larissa, University of Thessaly, 41110 Larissa, Greece; 3 Second Department of Dermatology-Venereology, “Attikon” General University Hospital, Medical School, National and Kapodistrian University of Athens, 12462 Athens, Greece

**Keywords:** plaque psoriasis, pregnancy, biologics in pregnancy, biologics, biosimilars, anti-TNF, adalimumab, certolizumab, ustekinumab

## Abstract

**Background:** Psoriasis in pregnancy is a highly prevalent inflammatory condition worldwide, with patients asking how the disease itself, as well as the medications that they take, would affect the general health of the fetus and the course of pregnancy. **Objectives:** This narrative review aims to explore the effects of biologic efficacy and safety on the pregnancy of women with psoriasis and on their fetus. **Methods:** A literature search based on biologic therapy, plaque psoriasis and pregnancy terms was conducted. Eleven articles were chosen after screening based on the inclusion and exclusion criteria. **Results:** The study recorded 290 pregnancies with psoriasis on biologics across 11 case series, resulting in 211 healthy live births (approximately 72.7% of pregnancies). Ustekinumab is the most extensively studied biologic therapy during pregnancy, with 241 reported pregnancy cases, compared with 42 cases documented for anti-TNF therapies. Most of the biologics were paused early in pregnancy, while 24 pregnancy cases under biologic treatment throughout pregnancy were detected. Most of those cases were under Certolizumab and Adalimumab in a percentage of 33.3% each. Among the 290 biologic-exposed pregnancies included in the analysis, 33 spontaneous abortions, 20 elective terminations, 17 preterm births, four stillbirths, eight low-birth-weight infants, and four congenital anomalies (three unspecified and one case of fetal exencephaly) were reported, but without biologic exposure timing and biologic-specific correlations. Certolizumab was considered to be safe throughout pregnancy with minimal incidence of adverse effects, in accordance with existing guidelines. **Conclusions:** Current evidence indicates that biologic therapy use during pregnancy among women with psoriasis is generally associated with favorable maternal and fetal outcomes. Reported rates of miscarriage, congenital anomalies, and live births appear to be comparable with those observed in the general population and in other inflammatory conditions. Further large-scale, prospective registry studies are needed to clarify whether specific biologic treatment approaches influence pregnancy outcomes and to support more evidence-based clinical decision-making.

## 1. Introduction

Psoriasis is a chronic immune-mediated inflammatory disorder that frequently affects women of reproductive age, making disease management during pregnancy an important clinical consideration. Pregnancy influences the clinical course of psoriasis through complex hormonal and immunological changes. Approximately 55% of women experience an improvement in disease activity during pregnancy, whereas around 23% report disease worsening. In the postpartum period, 65% of patients experienced worsening of their psoriasis [1]. Those changes are largely attributed to the physiological shift from a predominantly Th1/Th17-mediated immune response toward a Th2-dominant state, which promotes maternal immune tolerance of the developing fetus. Pregnancy hormones play a central role in mediating this immunological adaptation [2].

Despite this tendency toward disease improvement during pregnancy, uncontrolled or moderate-to-severe psoriasis has been associated with adverse maternal and fetal outcomes. The psoriasis-induced chronic systemic inflammation, together with its associated comorbidities, including diabetes, cardiovascular disease, and depression, may contribute to an increased risk of adverse pregnancy outcomes. Based on a Swedish and Danish national cohort study, women with psoriasis have been reported to have an increased risk of gestational diabetes, gestational hypertension, pre-eclampsia, and both elective and emergency cesarean delivery. Women with severe psoriasis were also found to have a higher risk of preterm birth, premature rupture of membranes, and delivering infants with low birth weight [3,4]. Psoriasis and ectopic pregnancy have also been correlated [1].

Conversely, other studies have reported no significant association between psoriasis and adverse pregnancy outcomes, with the spontaneous abortion percentage remaining the same as with the control group [1]. Worth mentioning is that women with high-severity psoriasis had a lower observed pregnancy rate compared with those receiving low-severity treatment; however, the difference was not statistically significant [5]. Interestingly, there was no multi-participant study with exact PASI score associations, only case reports [6]. The findings highlight the complexity of assessing the impact of psoriasis on pregnancy outcomes, as disease severity, comorbidities and treatment exposure create a vicious circle that influences observed risks.

The PSOLAR study, which assessed pregnancy outcomes in psoriasis and treatment status, found that women with psoriasis who had never been treated within one year had spontaneous abortion, a proportion of 13% compared to 16.7% in the case of biologic treatment during the prenatal period and 8.7% outside the prenatal period. However, the exact correlation cannot be concluded due to the limited number of participants and the fact that women who continued biologic therapy during pregnancy were more likely to have moderate-to-severe psoriasis and therefore a higher systemic inflammatory burden than those who discontinued biologics or had never received them. Consequently, in this study, any differences in pregnancy outcomes may reflect the effects of uncontrolled systemic inflammation rather than biologic or general treatment exposure itself [7].

Compared to psoriasis, achieving prompt control of disease activity during pregnancy is a primary therapeutic objective in inflammatory disorders such as inflammatory bowel disease (IBD). Active maternal disease is associated with a substantially greater risk of adverse pregnancy outcomes than the therapies used to induce and maintain remission. Consequently, pregnant women with active IBD should generally be managed according to the same therapeutic principles as non-pregnant patients. Effective treatments, including corticosteroids, anti-TNF agents, vedolizumab, and ustekinumab, should not be withheld solely because of pregnancy when clinically indicated, as maintaining disease remission is the most important predictor of favorable maternal and fetal outcomes [8].

Although systemic treatments are generally recommended to be discontinued before conception, management should be individualized according to disease severity and the risks and benefits for both the mother and fetus. When systemic therapy is required to maintain disease control, tumor necrosis factor-alpha (TNF-α) inhibitors such as certolizumab pegol (CZP) and cyclosporine are considered appropriate treatment options during pregnancy [9]. According to the last update of the EuroGuiDerm guideline for the treatment of psoriasis vulgaris systemic treatment, CZP is suggested as the first-line choice when starting biologic therapy in women planning conception (when a biologic is considered essential to use in pregnancy) and when it is necessary to start a systemic therapy during the second or third trimester. That suggestion is based on the fact that CZP is an Fc-free biologic; it does not bind the neonatal Fc receptor, which is responsible for active transplacental transport of biologic molecules developed on syncytiotrophoblast following 14 weeks of gestation [10].

In the case of anti-TNF biologics except CZP, they are recommended to be discontinued around 20 weeks’ gestation [11]. After this point, they increasingly cross the placenta during the second and third trimesters, leading to higher drug exposure in the fetus. As a result, the baby’s immune response may be temporarily reduced; therefore, live vaccines should be postponed until the infant is at least 6 months old. Also, there are specific anti-TNF-alpha biologic considerations. For example, Etanercept crosses the placenta to a much lesser extent because of its molecular structure and CZP does not cross the placenta, allowing it to be continued throughout pregnancy when clinically indicated. Additionally, women receiving certolizumab pegol are encouraged to breastfeed as very small amounts get to the newborn child via breast milk [12]. This approach primarily reflects concern regarding increasing placental transfer and neonatal drug exposure rather than demonstrated fetal toxicity.

There is less evidence regarding the exposure of other biologic agents, such as ustekinumab, an interleukin (IL)-12/23 inhibitor. The available data are limited and are based primarily on observational studies, case reports and pregnancy registries, while no standard biologic-specific risk of major congenital malformations, spontaneous abortion, preterm birth, low birth weight, or other adverse maternal or neonatal outcomes have been consistently demonstrated. Therefore, the present narrative review aims to evaluate all currently available data on the use of biologic therapies during pregnancy in women with psoriasis, with a particular focus on maternal, fetal, and neonatal outcomes, in order to provide an up-to-date overview of the current evidence and identify remaining knowledge gaps.

## 2. Results

This review included 11 studies, of which seven were case series, three were case reports and one was a retrospective observational registry. The total number of patients who took biologics for chronic plaque psoriasis during pregnancy was 290 (Table 1 and Table 2).

Across the 11 case series in the study, 290 pregnancies in women with psoriasis receiving biologic therapy were recorded, resulting in 211 healthy live births, accounting for approximately 72.7% of pregnancies. By excluding the multi-subject study of Mahadevan U et al. [23], we reported 71 pregnancies in women with psoriasis under biologic treatment with 53 healthy live births (74.6%). In both calculations, seven pregnancies also reported overlap with psoriatic arthritis. The mean age at pregnancy was reported to be 31.7 years. Ethnicity was recorded in only one of the case studies [17].

Most reports included anti-TNF drugs (10 out of 11 studies), with ustekinumab addressed in eight studies, secukinumab and guselkumab in two of the studies each. Infliximab was the most commonly used anti-TNF, followed by certolizumab, then etanercept, based on their frequency of use, as shown in Table 2.

Most pregnancies did not have a baseline PASI score recorded, except in four studies. Most of these pregnancies involved initiation of biologic treatment prior to conception, with remission of the disease at the time of pregnancy. Cases explicitly reported the scoring for each case over the course of monitoring, reflecting the severity of the disease. Some pregnancies showed worsening psoriasis towards the later part of gestation [6,16,20]. Psoriasis worsened in 22 out of 71 (30.98%) pregnancies, with PASI scores ranging from 5 to 26.5. All of these pregnancies paused biologic treatment during the first trimester. Boggs J.M. et al. exclusively noted that six out of nine pregnancies reported exacerbation of psoriasis following cessation of biologics [13].

In the review, 24 pregnancies out of 71 (33.8%) involved continuous biologic use, while the majority discontinued early in pregnancy. From these, 15 pregnancies (62.5%) resulted in live births, while six cases ended in miscarriages (25%), two (8.3%) in elective termination, and one (4.16%) in stillbirth. Adalimumab and CZP accounted for the largest proportion of continuous exposures (33.3%) of cases reported, followed by infliximab and ustekinumab (12.5% each), and secukinumab and guselkumab (4.2% each).

Among the reviewed pregnancies, one case reported cessation of biologic therapy three months before conception and one case reported by Espositio and colleagues initiated CZP at 24 weeks of gestation [16]. The group that stopped treatment early experienced more adverse outcomes—two spontaneous abortions, three elective terminations, one ectopic pregnancy, three cases of preterm labor, and one low-birth-weight delivery. Mugheddu et al. showed that stopping biologics is temporally linked to psoriasis worsening, which can lead to preterm labor and low birth weight [6]. Among the 24 pregnancies continuing medication, CZP was deemed safe, with six healthy outcomes reported. There was one stillbirth and one miscarriage in this group; the stillbirth was likely due to Down syndrome, diagnosed postmortem, with the previous miscarriage occurring while on ciclosporin. The cause for miscarriage is unclear.

The reported pregnancy outcomes were broadly similar regardless of whether the study by Mahadevan et al. was included. When all studies, including Mahadevan et al., were analyzed, the miscarriage rate was 11.3% (33/290), the elective termination rate was 6.8% (20/290), and the healthy live birth rate was 72.7% (211/290). Excluding the Mahadevan et al. study [22], the miscarriage rate remained virtually unchanged at 11.2% (8/71), while the elective termination rate increased to 9.8% and the healthy live birth rate increased to 74.6%. Daham et al. documented a case of fetal exencephaly that resulted in a medical termination [14]. The reasons for the other terminations were not related to the detection of anomalies and remain unspecified. A total of 33 miscarriages were recorded, and more specifically 28 cases with ustekinumab, followed by Adalimumab (two), secukinumab (two) and CZP (one), in descending order. These represent absolute event counts and should not be interpreted as comparative incidence or risk between biologic therapies, as the number of exposed pregnancies differed substantially across biologic agents. Five miscarriages were recorded in pregnancies that continued on biologics throughout, raising suspicion of drug-induced causation. However, there was no concrete evidence in these cases to establish causality.

One of the cohort studies is a major contributor to this review, accounting for more than half of the sample size, and it exclusively used Ustekinumab as the biologic [22]. The study clearly reported two subsets of the population receiving Ustekinumab at two different doses—45 mg and 90 mg. The results showed a higher percentage of adverse outcomes in the lower-dose group (33.8%) compared with the higher-dose group (27.2%). In addition, elective termination, stillbirth, spontaneous abortion, congenital anomalies, and other adverse events were well recorded. However, it cannot be ascertained that these outcomes are specific to Ustekinumab.

Stillbirth, low birth weight and congenital anomaly incidences among the neonates were also reported. The incidence of adverse fetal outcomes was highest among patients who received Ustekinumab [22]. Low birth weight is not an uncommon association reported with moderate-to-severe psoriasis. The case report by Mugeddu and colleagues demonstrated low birth weight in the newborn resulting from exacerbation of psoriasis rather than from the biologic, which was paused early in pregnancy, while Mahadevan et al. reported seven cases of low birth weight as one of the adverse outcomes [6].

Daham N et al. reported a case of a pregnant patient on Adalimumab who was diagnosed with a congenital anomaly of fetal exencephaly. The mother also had a long-standing history of psoriasis since the age of four, a history of an upper respiratory chest infection, and received Amoxicillin and clavulanic acid for treatment in the first trimester. She did not receive folic acid supplements during pregnancy and was known to have a higher BMI. The pregnancy was medically terminated [14]. The rest of the major congenital anomalies were recorded in the retrospective cohort [22]. The pattern of anomalies reported in this particular study did not demonstrate any specific or consistent characterization. Given the broad terminology used by the authors to describe adverse outcomes, three unspecified cases were specifically classified as major congenital defects. There was no incidence of neonatal infections in this cohort of case series. Respiratory distress syndrome was one of the only fetal complications noted. There was no established sepsis in the neonates. Mahadevan et al. reported several neonatal adverse outcomes such as amniotic cavity infection, jaundice, cardio respiratory arrest, fetal growth restriction, fetal hypokinesia, infection, neonatal respiratory distress and penile cellulitis [22].

## 3. Discussion

According to our review, approximately 72.7% of pregnancies of women with psoriasis exposed to biologic therapy resulted in live births without reported abnormalities or complications; however, this finding should be interpreted cautiously. Much of the available evidence reported derives from case reports, case series, and pharmacovigilance registries, which are susceptible to reporting bias as adverse or unusual pregnancy outcomes are more likely to be reported than uncomplicated pregnancies, whereas normal deliveries may remain unpublished or underrepresented. Consequently, the observed frequency of normal births may not accurately reflect the true risk profile, and the incidence of adverse outcomes may be either over- or underestimated. Compared with the mixed psoriasis population of PSOLAR (women with psoriasis, including those who were under biologics), 81.9% resulted in uneventful births, 13.8% ended in spontaneous abortion, and 4.4% were electively terminated [7]. In the case of our review, by excluding the multi-participant study of Mahadevan U et al. [22] to better assess the percentages of multi-biologic effects on pregnancy, the exact percentages were 74.7% in uneventful births, 9.9% in spontaneous abortions and 6.8% elective termination. This concordance in both studies suggests that biologic exposure is not associated with a substantially different distribution of pregnancy outcomes. Also, in the PSOLAR study, 93.1% of women under biologic therapy during the prenatal period gave birth to a normal child while 16.7% of this pattern of pregnancies led to spontaneous abortions. In the case that the biologic therapy exposure occurred outside the prenatal period, the normal birth percentage improves slightly to 94.7% and spontaneous abortion rate falls to 8.3% (almost half of the previously mentioned percentage), suggesting that timing of biologic therapy exposure may play an important role in pregnancy outcomes [7]. In our review, several pregnancies occurred in women who were receiving biologic therapy and were not using contraception, resulting in unplanned conceptions. In many cases, pregnancy was recognized only after inadvertent first-trimester exposure had already occurred. Following pregnancy confirmation, biologic therapy was frequently discontinued; however, in some women, psoriasis worsened after treatment cessation, necessitating continuation or reintroduction of the biologic. Consequently, first-trimester exposure was the most common pattern of biologic exposure observed and was seen across all biologics (adalimumab, ustekinumab, secukinumab, infliximab, etanercept, and guselkumab). After excluding the Mahadevan et al. study, large Ustekinumab cohort [22], continuous biologic exposure throughout pregnancy was reported in 24 pregnancies (33.8%). From these, 15 pregnancies (62.5%) resulted in live births, while six cases ended in miscarriages (25%), two (8.3%) in elective termination, and one (4.2%) in stillbirth. Adalimumab and CZP accounted for the largest proportion of continuous exposures (33.3% each) of cases reported, infliximab and ustekinumab (12.5% each) and secukinumab and guselkumab (4.2% each). Although adverse outcomes were observed across several biologic classes, no consistent association with a specific agent emerged. Most pregnancies exposed to infliximab and adalimumab throughout gestation resulted in healthy live births, whereas the few pregnancy losses reported with ustekinumab, secukinumab, certolizumab pegol, and guselkumab occurred as isolated cases. Given the small number of continuously exposed pregnancies and the predominance of case reports and case series, these findings do not support biologic-specific safety signals.

**Figure 1:**
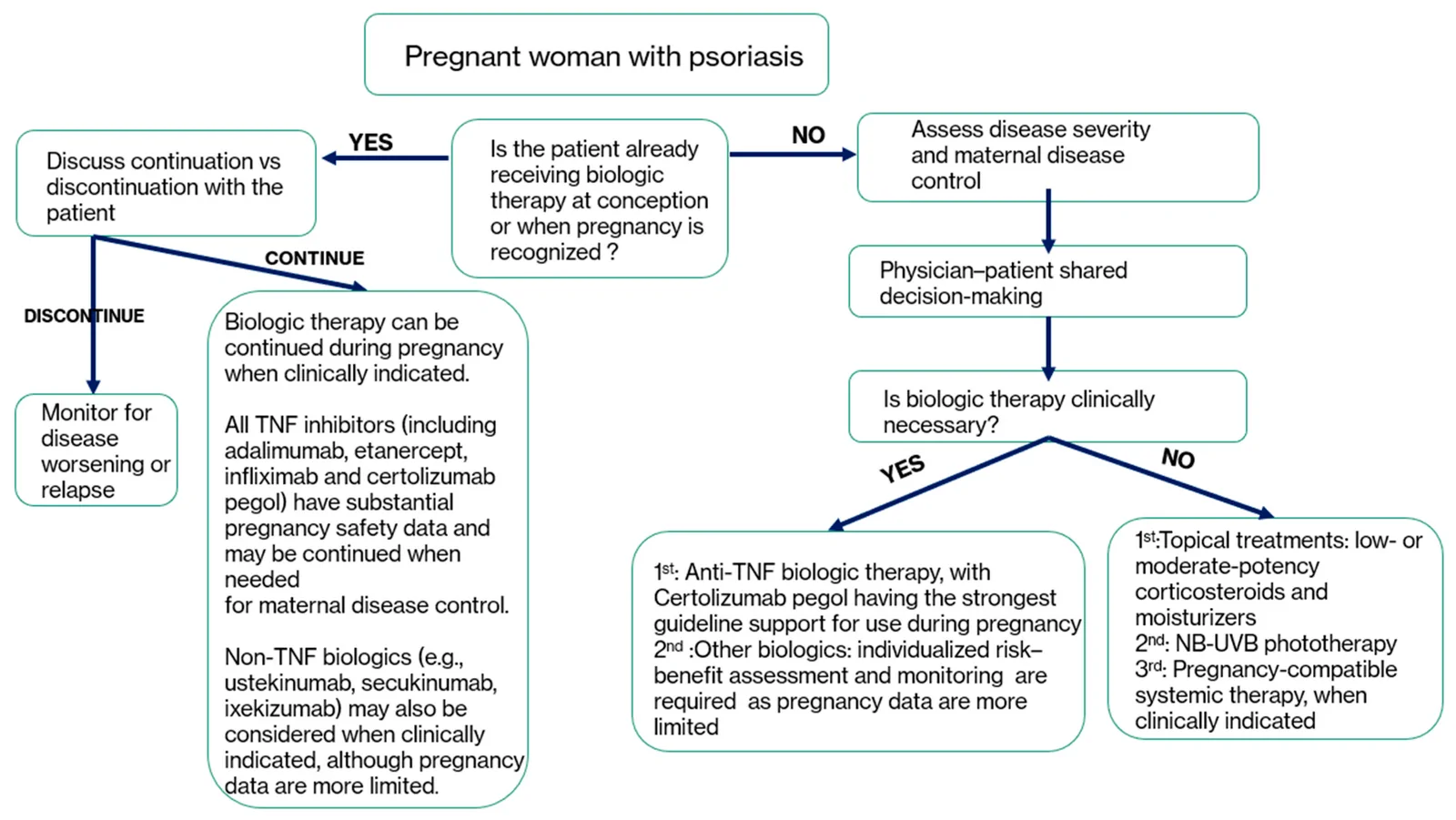
Physician–patient decision-making pathway for the management of psoriasis during pregnancy.

In the case of the large biologic-specific study on ustekinumab, 76.4% of women with psoriasis under 45 mg Ustekinumab and 89.1% under 90 mg reported normal births, suggesting that higher dose is not connected with higher risk of unwanted pregnancy outcomes. Regarding a systemic review that focused on biologics used in psoriasis beyond plaque subtype, 75.0% of pregnancies resulted in live births, while 14.5% ended in spontaneous abortion and 10.3% in elective termination. Among reported live-birth outcomes, 2.8% of infants had congenital malformations, and 7.9% were small for gestational age. These findings are broadly comparable to those observed in our review, supporting the growing body of evidence that biologic exposure during pregnancy is not associated with a substantial increase in adverse pregnancy [22]. Moreover, the reported prevalence of miscarriages and reported congenital malformations in the general obstetric population [23] is similar to that observed among pregnancies exposed to biologic therapy. Additionally, the findings of our review are consistent with the medical literature existing on inflammatory bowel disease, where biologic therapy during pregnancy has been evaluated in substantially larger prospective cohorts and meta-analyses. For example, in the largest meta-analysis to date, including 6963 biologic-exposed pregnancies, no increased risk associated with continuation of anti-TNF therapy throughout pregnancy was reported [24]. Likewise, a meta-analysis of 1216 pregnancies found no significant increase in spontaneous abortion, preterm birth, low birth weight, or congenital malformations among women receiving anti-TNF therapy compared with unexposed controls. These findings closely parallel those observed in our review, in which 72.7% of pregnancies resulted in uneventful live births, 9.9% ended in spontaneous abortion, and congenital anomalies were uncommon [25]. Collectively, the evidence from psoriasis and IBD suggests that maintaining maternal disease control with biologic therapy may be more important for optimizing pregnancy outcomes than concerns regarding biologic exposure itself, although disease-specific prospective registries remain necessary to confirm these observations (Table 3, Figure 1).

Regardless of medical specialty and according to the updated EULAR recommendations, the evidence supporting the safety of biologic disease-modifying antirheumatic drugs during pregnancy has expanded considerably. In particular, accumulating data, including recent meta-analytic evidence, indicate that anti-TNF inhibitor biologics, including infliximab, adalimumab, golimumab, etanercept and CZP, are not associated with an increased risk of congenital malformations, miscarriage, or other adverse pregnancy outcomes. Furthermore, in utero exposure to anti-TNF biologic therapy, including exposure during the third trimester, has not been associated with an increased risk of serious infections in infants during the first year of life. Because placental transfer of certain biologics during the second half of pregnancy may result in detectable neonatal drug levels and influence the timing of live vaccines, EULAR proposes treatment-specific strategies to minimize fetal exposure. Whole IgG1 monoclonal antibodies such as infliximab may be discontinued by gestational week 20, whereas Fc-fusion proteins such as etanercept may be stopped at approximately weeks 30–32. In contrast, certolizumab pegol, which lacks an Fc region and consequently has minimal placental transfer, can be continued throughout pregnancy when clinically indicated. When required to maintain adequate maternal disease control, several non-TNFi biologics—including abatacept, anakinra, belimumab, canakinumab, ixekizumab, rituximab, sarilumab, secukinumab, tocilizumab, and ustekinumab—may also be considered during pregnancy. Available evidence does not suggest an increased frequency of adverse pregnancy outcomes with these agents compared with background rates in the general population, although the level of evidence remains substantially more limited than that available for TNFi. Conversely, agents for which pregnancy safety data remain insufficient—including apremilast, avacopan, baricitinib, bosentan, filgotinib, leflunomide, mepacrine, tofacitinib, upadacitinib, and voclosporin—should generally be avoided during pregnancy or replaced with pregnancy-compatible therapies when pregnancy is planned. Importantly, this recommendation reflects insufficient evidence of safety rather than established evidence of fetal harm [26].

This review has several limitations related to the structure of the available evidence. The majority of included studies were case reports, case series, and small observational cohorts, limiting the ability to establish causal relationships between biologic exposure and pregnancy outcomes. These study designs are particularly vulnerable to publication and reporting bias, as adverse outcomes may be preferentially reported. Significant heterogeneity was observed regarding patient characteristics, psoriasis severity, presence of psoriatic arthritis, biologic class, exposure timing, treatment duration, and outcome definitions, limiting direct comparisons between studies. Furthermore, information regarding cumulative biologic dose, disease activity during pregnancy, concomitant treatments, and maternal risk factors was frequently incomplete, preventing adjustment for potential confounding factors. The limitations of the review process also include the use of a single database, restriction to English-language publications and the inclusion mainly of case reports and case series. The relatively small number of pregnancies exposed to individual biologic agents, particularly newer IL-17 and IL-23 inhibitors, limits the ability to draw drug-specific safety conclusions. Therefore, although the findings are reassuring and consistent with larger pregnancy datasets from other inflammatory diseases, they should be interpreted cautiously and require confirmation through larger prospective registries with standardized outcome reporting.

## 4. Materials and Methods

A narrative review was conducted to evaluate the available evidence on the use of biologic therapies for chronic plaque psoriasis during pregnancy. A comprehensive literature search was performed in PubMed to identify relevant studies published between January 2016 and January 2026. The search strategy combined Medical Subject Headings (MeSH) and free-text keywords related to psoriasis, pregnancy, and biologic therapy using Boolean operators (AND/OR). Search terms included a combination of plaque psoriasis, pregnancy, and biologics, such as anti-TNF, adalimumab, etanercept, certolizumab and secukinumab. This review focused on real-world evidence derived from case reports, case series, pregnancy registries, and observational studies. English-based studies involving pregnant women with chronic plaque psoriasis receiving biologic therapy were eligible for inclusion. Patients with psoriasis arthritis only without skin manifestations or under any other systemic anti-inflammatory treatment were excluded. The findings were synthesized narratively and organized into thematic sections addressing the impact of psoriasis during pregnancy, the safety and effectiveness of individual biologic agents, maternal and fetal outcomes, neonatal and long-term infant health, and reported adverse events.

## 5. Conclusions

In conclusion, the available evidence suggests that biologic therapy during pregnancy in women with psoriasis has not been associated with a clear increase in adverse maternal or fetal outcomes, with rates of miscarriage, congenital malformations, and live births comparable to those reported in the general population and other inflammatory diseases. Current clinical guidelines and recommendations support the use of anti-TNF biologics, particularly certolizumab pegol, during pregnancy, while evidence for other biologics, including ustekinumab, secukinumab, and guselkumab, remains more limited. Across the included studies, no specific biologic agent, timing of exposure, or treatment continuation pattern appeared to be consistently associated with an increased risk of adverse pregnancy outcomes. Larger prospective registries are required to determine whether specific biologic practices influence pregnancy outcomes and to provide more precise guidance for clinical decision-making.

## Figures and Tables

**Table 1:** Table summarizing pregnancy outcomes in women with psoriasis under biologic therapy.

Study	Pregnancy Cases	Biologic Used	PsA Co-Existence	Biologic Exposure During Pregnancy	Psoriasis Severity During Pregnancy	Maternal Outcome	Pregnancy Outcome	Fetal Outcome (Ps)	Fetal Outcome (Ps and PsA)
[13]	5	ADA	Present in 2 of them	Periconception period only	Exacerbation reported	No further complications mentioned	Uneventful	Healthy children	-
	4	ADA	Present in 1 of them	Throughout pregnancy	NM	No further complications mentioned	Uneventful in the rest; 1 miscarriage	Healthy children	Miscarriage
	1	ETA	Present	Periconception period only	NM	No further complications mentioned	Uneventful	Healthy child	-
	1	SEC	Present	Throughout pregnancy	NM	No further complications mentioned	Miscarriage	-	Miscarriage
	1	SEC	Present	Periconception period only	Exacerbation reported	No further complications mentioned	Miscarriage	-	Miscarriage
[14]	1	ADA	No/NM	Throughout pregnancy	No/NM	No further complication	Elective termination at 13 weeks	Congenital defect (fetal acrania- exencephaly)	-
[15]	1	ADA	No/NM	Periconceptional—paused at week 4	No/NM	No further complications mentioned	Uneventful	Healthy child	-
	1	ADA	No/NM	Periconceptional—paused at week 12	No/NM	Systemic hypertension	Uneventful	Respiratory distress in newborn	-
	1	ETA	No/NM	Periconceptional—paused at week 4	No/NM	No further complications mentioned	Uneventful	Healthy child	-
	1	ETA	No/NM	Periconceptional—paused at week 5	No/NM	Sciatic pain, erythema nodosum, nausea, asthenia, dizziness and vaginal infection	Detachment of amniotic sac	Healthy child	
	1	ETA	No/NM	Periconceptional—paused at week 4	No/NM	No further complications mentioned	Induced abortion (previous history of Down syndrome)	-	-
	1	UST	No/NM	Paused at week 16	No/NM	No further complications mentioned	Uneventful	Healthy child	-
[16]	2	CZP	No/NM	G1: Periconception period and during pregnancy (restarted 24 weeks of gestation); G2: Throughout pregnancy	Exacerbation with biologic cessation	No further complications mentioned	Elective cesarean section	Healthy	-
[17]	2	UST	No/NM	G1:Periconception period (stopped at week 5); G2: Periconceptional (paused at week 4)	Exacerbation reported in both pregnancies	No further complications mentioned	Uneventful	Healthy children	-
	1	UST	No/NM	Periconceptional	NM	No further complications mentioned	Uneventful	Healthy child	-
	1	UST	No/NM	Periconception period—paused at 4 months of gestation	Exacerbation reported	No further complications mentioned	Uneventful	Healthy child	-
[18]	2	IFX	Present	Periconceptional (paused at first trimester)	NM	Hepatic steatosis in 1 of them. PCOS, DM, hyperlipidemia, obesity, hepatosteatosis and smoking in the other	Uneventful		Healthy
	1	ETA	NM	Periconceptional (paused at first trimester)	NM	No further complications mentioned	Uneventful	Healthy	-
	1	UST	NM	Periconceptional (first trimester)	NM	No further complications mentioned	Uneventful		
	1	UST	NM	Periconceptional (first trimester)	NM	No further complications mentioned	Right tubal pregnancy. Terminated at 8 weeks		
	1	ADA	Present	Periconceptional (first trimester)	NM	Hepatic fibrosis,	Elective termination	-	
[19]	1	IFX	No	Throughout pregnancy	No	Elevated aspartate aminotransferase and Lactate dehydrogenase in this pregnancy (attributed to paracetamol)	Uneventful	Healthy	-
	1	IFX	No	Throughout pregnancy	No	No further complications mentioned	Uneventful	Healthy twins	-
	1	IFX	No	Paused during First trimester	No	No further complications mentioned	Uneventful	Healthy	-
	1	IFX	No	Throughout pregnancy	No	No further complications mentioned	Uneventful	Healthy	-
	1	UST	No	Periconceptional—paused 3 months before conception	No	Hydronephrosis (genetically predisposed)	Manual removal of placenta following natural birth	Healthy	-
	1	UST	No	Periconceptional—paused at first trimester	No	No further complications mentioned	Uneventful	Healthy	-
	1	UST	No	Periconceptional—paused at first trimester	No	No further complications mentioned	Uneventful	Healthy	-
	1	ADA	No	Throughout pregnancy	No	Elevated ALP, anemia, (due to pregnancy)	Uneventful	Healthy	-
	1	ADA	No	Throughout pregnancy	No	Elevated ALP, anemia, (due to pregnancy)	Uneventful	Healthy	-
[6]	1	ADA	No	Periconceptional—paused at 8th wk	Exacerbation reported (PASI 18)	No further complications mentioned	Preterm labor	Healthy	-
	1	UST	No	Periconceptional—paused at 6th wk	Exacerbation reported (PASI 15)	No further complications mentioned	Preterm labor	Low birth weight	-
	1	UST	No	Periconceptional—exact time of pause not documented	Exacerbation reported (PASI 17.4)	No further complications mentioned	Preterm labor	Healthy	-
[20]	1	SEC	No	Periconceptional—paused at 4th wk	Exacerbation reported	No further complications mentioned	Uneventful	Healthy	-
	1	SEC	No	Periconceptional—paused at 5th wk	Exacerbation reported	No further complications mentioned	Uneventful	Healthy	
	1	IFX	No	Periconceptional—paused at 3rd wk	Exacerbation reported	No further complications mentioned	Uneventful	Healthy	
	1	IFX	No	Periconceptional—paused at 6th wk	No	No further complications mentioned	Elective abortion	-	-
	1	IFX	No	Periconceptional—paused at 6th wk	Exacerbation reported	No further complications mentioned	Uneventful	Healthy	
	1	UST	No	Periconceptional—paused at 6th wk	Exacerbation reported	No further complications mentioned	Uneventful	Healthy	
	1	IFX (CZP started midway)	No	Periconceptional—paused at 6th wk	Exacerbation reported	No further complications mentioned	Uneventful	Healthy	
	1	ETA	No	Periconceptional—paused at 6th wk	No	No further complications mentioned	Uneventful	Healthy	
	1	UST	No	Periconceptional—paused at 6th wk	Exacerbation reported	No further complications mentioned	Uneventful	Healthy	
	1	ADA	No	Periconceptional—paused at 5th wk	Exacerbation reported	No further complications mentioned	Uneventful	Healthy	
	1	UST	No	Periconceptional—paused at 8th wk	Exacerbation reported	No further complications mentioned	Uneventful	Healthy	-
	1	UST	No	Periconceptional—paused at 6th wk	No	No further complications mentioned	Spontaneous abortion	-	
	1	UST	No	Periconceptional—paused at 6th wk	No	No further complications mentioned	Elective abortion	-	-
[21]	4	CZP	No	Throughout pregnancy	NM	No further complications mentioned	Uneventful	Healthy	
	1	CZP	No	Throughout Pregnancy	NM	No further complications mentioned	Miscarriage	-	
	1	CZP	No	Throughout pregnancy	NM	No further complications mentioned	Uneventful	-Stillbirth	
	1	GSK	No	Periconceptional—paused at 7th wk	NM	No further complications mentioned	Uneventful	Healthy	
	1	GSK	No	Periconceptional—paused at 15th wk	NM	No further complications mentioned	Uneventful	Healthy	
	1	GSK	No	Throughout pregnancy	NM	No further complications mentioned	Elective termination	-	
	1	ADA	No	Throughout pregnancy	NM	No further complications mentioned	Miscarriage	-	
	1	UST	No	Throughout pregnancy	NM	No further complications mentioned	Miscarriage	-	
	1	UST	No	Throughout pregnancy	NM	No further complications mentioned	Miscarriage	-	
	1	UST	No	Periconceptional—paused at 8th wk	NM	No further complications mentioned	Uneventful	Healthy	
	1	UST	No	Throughout pregnancy	NM	No further complications mentioned	Uneventful	Healthy	
	1	UST	No	Periconceptional—paused at 2nd wk	NM	Uneventful	Uneventful	Healthy	
[22]	219	UST	No	Most of them are suspended in the first trimester, making it impractical to determine the exact time of biologic cessation	No/NM	No further complications mentioned	25 spontaneous abortions and 13 elective abortions 14 preterm births	158 healthy births, 3 congenital defects and anomalies and 3 still births; 7 low birth weight	

**Table 2:** Biologic-specific correlations of pregnancy courses.

Biologic (Total No of Pregnancies in This Study)	No of Spontaneous Abortion	No of Elective Terminations	Number of Healthy Births	Number of Preterm Labor	Number of Stillbirths	Number of Low Birth Weight in Neonates	Number of Major Congenital Anomalies
Adalimumab (18)	2	2	13	1	0	0	1
Etanercept (6)	0	1	5	0	0	0	0
Infliximab (10)	0	1	9	0	0	0	0
Ustekinumab (241/22 *)	28/3 *	15/2 *	174/16 *	16/2 *	3/0 *	8/1 *	3/0 *
CZP (8)	1	0	6	0	1	0	0
Guselkumab (3)	0	1	2	0	0	0	0
Secukinumab (4)	2	0	2	0	0	0	0
All (290/71 *)	33/8 *	20/7 *	211/53 *	17/3 *	4/1 *	8/1 *	4/1 *

* Number obtained by excluding the multi-participant study of Mahadevan U et al. [22].

**Table 3:** Main points on biologics, plaque psoriasis and pregnancy.

1—Available evidence indicates that biologic exposure is not associated with an increased risk of miscarriage, major congenital malformations, stillbirth, or other adverse neonatal outcomes, with reported rates remaining comparable to those observed in the general population. No consistent biologic-specific side effects were detected.
2—Clinical decision-making should continue to follow existing evidence-based recommendations. Among the available biologics, anti-TNF agents remain the best-supported therapies during pregnancy, with the strongest evidence supporting their safety. In particular, certolizumab pegol, owing to its minimal placental transfer, remains the preferred biologic choice, that can be continued throughout pregnancy.
3—Current data are insufficient to show that cumulative biologic dose or continuation of therapy throughout pregnancy or the timing of biologic exposure influence pregnancy outcomes.
4—During pregnancy, the decision to continue or discontinue biologic therapy should be individualized through shared physician–patient discussion. When biologic therapy is not selected, alternative treatments may include topical therapies, particularly mild-to-moderate-potency topical corticosteroids and moisturizers. As a second line, narrowband UVB phototherapy, which is not contraindicated during pregnancy, can be applied. In patients requiring systemic treatment, ciclosporin may be considered when clinically necessary, whereas methotrexate, fumarates and acitretin are contraindicated during pregnancy.
5—Discontinuation should not be routinely recommended solely because of pregnancy when the benefits of maintaining disease control outweigh potential risks. Evidence from psoriasis, inflammatory bowel disease, and other immune-mediated inflammatory disorders suggests that biologic treatments are considered safe during pregnancy and treatment decisions should prioritize individualized risk–benefit assessment. However, further prospective studies and pregnancy-specific safety data are needed, particularly for biologic agents with limited pregnancy exposure data.

## Data Availability

No new data were created or analyzed in this study. Data sharing is not applicable to this article.
